# Recall by polygenic risk score in two biobanks supports a genomic approach for glaucoma detection

**DOI:** 10.21203/rs.3.rs-7159368/v1

**Published:** 2025-08-05

**Authors:** Janey Wiggs, Louis Pasquale, Hetince Zhao, Nazlee Zebardast, Kanza Aziz, Yan Zhao, William Steidl, Jae Kang, James Tsai, Nicole Paulescu, Ghislain Rocheleau, Tobias Elze, Michael Boland, Ha My Vy, Ron Do, Ayellet Segrè, David Friedman

**Affiliations:** Icahn School of Medicine at Mount Sinai; Department of Ophthalmology, Icahn School of Medicine at Mount Sinai, New York Eye and Ear Infirmary of Mount Sinai, New York, New York, USA; Massachusetts Eye and Ear; Massachusetts Eye and Ear; Massachusetts Eye and Ear; 1 Department of Ophthalmology, IcIcahn School of Medicine at Mount Sinai, New York Eye and Ear Infirmary of Mount Sinai; Brigham and Women’s Hospital / Harvard Medical School; Department of Ophthalmology, Icahn School of Medicine at Mount Sinai, New York Eye and Ear Infirmary of Mount Sinai, New York, New York, USA; Department of Ophthalmology, Icahn School of Medicine at Mount Sinai, New York Eye and Ear Infirmary of Mount Sinai, New York, New York, USA; Icahn School of Medicine at Mount Sinai; Massachusetts Eye and Ear; Department of Ophthalmology, Harvard Medical School, Massachusetts Eye and Ear Infirmary, Boston, Massachusetts, USA; Icahn School of Medicine at Mount Sinai; Icahn School of Medicine at Mount Sinai; Massachusetts Eye and Ear; Department of Ophthalmology, Harvard Medical School, Massachusetts Eye and Ear Infirmary, Boston, Massachusetts, USA

## Abstract

Glaucoma is a highly heritable optic neuropathy and a leading cause of blindness; yet, glaucoma screening is challenging due to the time-consuming clinical methods required for disease identification and the imperfect accuracy of screening tests. We used genome-wide association study results to calculate a primary open angle glaucoma (POAG) polygenic risk score (PRS) for Mount Sinai Bio*Me* and Mass General Brigham Biobank participants. Glaucoma prevalence for recalled individuals in the top and bottom PRS decile groups were compared after standardized clinical examinations masked to PRS status. The top PRS decile group had an overall glaucoma prevalence of 18.8% and was 6.7 times (Odds Ratio 95% confidence interval: 3.1 – 14.3) more likely to be diagnosed with glaucoma compared to the bottom PRS decile group. Notably, 47.1% of identified glaucoma cases in the high-risk group were previously undiagnosed. These results support using PRS testing to detect glaucoma and to identify patients for increased surveillance or preventative treatment.

Glaucoma is a progressive optic neuropathy that is a relatively common cause of irreversible vision loss, currently affecting 76 million people worldwide, with projections for 111.8 million affected by 2040^1^. Primary open-angle glaucoma (POAG) constitutes 75% of glaucoma cases globally and accounts for over 50% of glaucoma-related blindness^2^. Since glaucoma-related vision loss occurs gradually, and early-stage disease typically affects the peripheral visual field, the condition can remain unrecognized even after substantial permanent damage to the optic nerve has occurred. Population studies suggest that more than 50% of affected people are unaware of their diagnosis.^3^ As the disease progresses, glaucoma-related vision loss negatively affects quality of life, increases societal economic burden, and puts individuals at a higher risk for falls and decreased mobility even in its early stages^4^.

Randomized clinical trials indicate that lowering intraocular pressure (IOP) can delay disease onset and slow progression, making early-stage glaucoma detection a top priority to prevent subsequent vision loss^5^. However, systematic assessments conducted in 2013^6^ and 2022^7^ by the US Preventive Services Task Force, considering benefits and harms of case identification, found insufficient evidence to recommend glaucoma screening.

Glaucoma is a highly heritable disease^8^ with more than 300 disease-associated loci discovered through genome-wide association studies (GWAS)^9,10^. Using this genetic information to identify high-risk individuals may allow for targeted screening. Polygenic risk scores (PRSs) representing the aggregated genome-wide disease risk^11^ have been associated with POAG in various cohorts with estimates of disease prevalence as high as 7.6% in the top PRS deciles and area under the curve of up to 0.82^12,13^. Because PRS can also predict disease progression^14^, earlier treatment escalation^15^, and the need for surgical intervention^15^, the strategic application of genetic risk assessment could strengthen the rationale for implementing glaucoma screening initiatives in clinical practice. Yet, true glaucoma prevalence in previous studies assessing PRS associations is unknown because only known cases were assessed, and undiagnosed cases could not be accounted for. To measure disease prevalence in high and low PRS groups, we calculated a POAG PRS for participants in two hospital-based biobanks and invited those in the top and bottom deciles for comprehensive clinical examination.

Using summary statistics from a large cross-ancestry GWAS meta-analysis^9^, a genome-wide POAG PRS was calculated (summary statistics available at https://segrelab.meei.harvard.edu/data/). The PRS was trained using imputed genotype data from UK Biobank participants by applying a Lassosum penalized regression framework^16^, as previously described^12^. A total of 144,009 single-nucleotide polymorphisms (SNPs) with non-zero beta values remained across the autosomes and chromosome X variants in the non-pseudoautosomal region after applying Lassosum regression. The calculated weights for these SNPs were used to compute a PRS for 36,217 participants in the Mass General Brigham (MGB) Biobank and 53,499 participants in the Bio*Me* Biobank using the “--score” function in PLINK (Supplementary Figure 1). Individuals 35–90 years of age in the top and bottom ancestry-standardized deciles were invited to enroll in the study. We included participants of European (EUR), African (AFR), Mixed American (AMR), and Asian (ASN) ancestry and did not exclude for any systemic conditions or family history of glaucoma.

235 individuals from the Bio*Me* Biobank and 284 individuals from the MGB Biobank (Supplementary Figure 1) underwent detailed standardized clinical examinations (Supplementary Figure 2) including measurement of visual acuity, IOP, slit lamp biomicroscopy, biometry, and gonioscopy. Optical coherence tomography (OCT) of the optic nerve and circumpapillary retina, fundus photographs, and visual field testing (Supplementary methods) were also performed. Glaucoma status (definitions in supplementary methods) was determined by the examining glaucoma specialist and confirmed by a second glaucoma-trained ophthalmologist. If the two disagreed, a third specialist resolved the diagnosis. The PRS was revealed only after a diagnosis had been established. Demographic features (Supplementary Table 1) were similar for the highest and lowest decile PRS groups, including age and ancestry. In Bio*Me*, the average age was 65.1 years (Standard Deviation (SD) =7.41), and genetically inferred ancestry was 36.6% EUR, 34.5% AFR, 24.7% AMR, and 4.3% ASN (Supplementary Table 1a). The average age was 64.6 years (SD=12.0) in MGB Biobank with 84.2% EUR, 8.8% AFR, 4.2% AMR, and 2.8% ASN (Supplementary Table 1b).

In Bio*Me*, probable or definite glaucoma was present in 27/119 (22.7%) participants in the top PRS decile group and 4/116 (3.5%) in the bottom decile group (Table 1). Similar results were observed in the MGB Biobank (15.6% vs. 4.6%). For the two biobanks combined, glaucoma was present in 51/273 (18.7%) participants in the top PRS group and 10/246 (4.1%) in the bottom PRS group (Table 1), an increase of nearly 10-fold over the reported average prevalence^2,3^. The overall odds ratio (OR) for glaucoma (probable/definite) in the top vs. bottom PRS groups was 6.7 (95% confidence interval (CI): 3.2 – 14.4) using a multiple logistic regression model, adjusted for age, sex, body mass index, hypertension, diabetes, axial length, central corneal thickness, and categorical genetically inferred ancestry. The OR for the African ancestry subgroup was 7.2 (95% CI: 1.7 – 30.1) (Table 1). Other ancestry groups were too small for subgroup analyses. Clinical features associated with glaucoma were also significantly different between the highest and lowest PRS decile groups (Table 2a), including IOP, retinal nerve fiber layer (RNFL) thickness, and visual field mean deviation (MD). These traits also exhibited expected trends among participants in the highest glaucoma PRS decile without clinical evidence of glaucoma versus those in the lowest PRS decile (Table 2b), suggesting an enrichment of individuals with increased preclinical glaucoma in the highest PRS decile.

This is the first study to invite participants with calculated PRS for a comprehensive ophthalmic examination, affording a unique opportunity to assess the utility of a genetic risk assessment instrument in detecting new glaucoma cases (i.e., a genotype-first, phenotype-second approach). Using participant interview information and available electronic health records, we determined that among individuals diagnosed with glaucoma, 49.2% were previously undiagnosed, including 47.1% of cases in the highest genetic risk group (Supplementary Table 2). This rate is consistent with previous population estimates of undiagnosed cases (estimated at approximately 50%)^3^, showing that a PRS screening approach can effectively detect known and unidentified disease.

In summary, these results, based on rigorous standardized clinical examinations, indicate that participants in the highest glaucoma PRS decile had a 6.7-fold higher disease prevalence compared to those in the lowest decile. The higher disease prevalence was observed for all sufficiently-sized ethnic groups included in the study, a particularly notable finding for African ancestry participants, as they are disproportionately affected by glaucoma^17^. The prevalence in the high-risk groups was higher than observed in studies based on diagnostic codes and self-report^12,13,18^ because we could identify and account for undiagnosed cases, an important outcome of this work. Importantly, the increase in prevalence in the high genetic-risk group supports the feasibility of targeted diagnostic testing using current clinical measures^19^.

A strength of our study is the use of two independent cohorts, allowing for replication of the findings. Limitations include the relatively small sample size, which yielded somewhat unstable, albeit significant, effect estimates for the relationship between PRS and glaucoma, especially for certain ethnic groups, potentially reducing the cross-ancestral applicability of the results. Additionally, this was a hospital-based, rather than a population-based, sample.

Overall, we demonstrate that individuals with high genetic risk are more likely to be affected by disease and that employment of PRS can be a useful glaucoma screening tool to identify new glaucoma cases in hospital-based DNA repositories. This work represents a significant step toward the clinical implementation of PRS tools and streamlined phenotyping strategies for detecting glaucoma cases that require treatment to prevent visual disability.

## Supplementary Material

Supplementary Files

This is a list of supplementary files associated with this preprint. Click to download.

- PRSscreeningsupplementaryR5.docx

## Figures and Tables

**Table 1.** Glaucoma prevalence in the highest and lowest polygenic risk score (PRS) decile groups and multivariable adjusted odds of glaucoma in the highest PRS decile group in two biobanks (N=519).

	Definite + Probable Glaucoma			
	High PRS decile	Low PRS decile	Multivariable-adjusted	P-value
	N/total (%)	N/total (%)	Odds Ratio (95% Confidence Interval)*	
**Bio*Me* (n=235)**	27/119 (22.7%)	4/116 (3.5%)	10.4 (3.2 – 33.8)	9.7 E-05
**MGBB (n=284)**	24/153 (15.7%)	6/131 (4.6%)	6.1 (1.9 – 19.4)	2.0 E-03
**Bio*Me*+MGBB (n=519)**	51/272 (18.8%)	10/247 (4.1%)	6.7 (3.1 – 14.3)	8.8 E-07
**EUR only (n=325)**	29/174 (16.7%)	3/150 (2.0%)	8.5 (2.4 – 29.8)	8.4 E-04
**AFR only (n=106)**	15/62 (24.2%)	4/45 (8.9%)	7.3 (1.7 – 30.3)	6.4 E-03
**AMR only (n=70)**	6/31 (19.4%)	3/39 (7.69%)	5.2 (0.9 – 30.2)	0.067
ASN only** **(n=18)**	1/5 (20.0%)	0/13 (0%)	–	–

* Multivariable-adjusted odds ratios estimated from multiple logistic regression models adjusted for continuous age, sex, body mass index, hypertension, diabetes, axial length, central corneal thickness, and categorical genetically inferred ancestry (only in multi-ancestry models).

** Insufficient sample size. Abbreviations: MGBB, Mass General Brigham Biobank; EUR, European; AFR, African; AMR, Mixed American; ASN, Asian.

**Table 2.** Clinical features of the highest decile PRS vs the lowest decile PRS individuals.

A. Including definite and probable glaucoma					
Trait	Highest PRS decile	Highest PRS decile (SE),	Lowest PRS decile	Lowest PRS decile (SE),	P-value
	N	95% CI	N	95% CI	
**Goldman IOP (mmHg)** ^1^	228	15.4 (0.3), 14.7 – 16.0	231	13.7 (0.3), 13.1 – 14.3	3.5 E-07
**Corneal compensated IOP (mmHg)** ^1^	229	16.6 (0.3), 16.0 – 17.3	233	14.9 (0.3), 14.3 – 15.5	5.5 E-07
**Global Circumpapillary RNFL thickness (μm)**	261	90.7 (1.2), 88.3 – 93.0	236	94.4 (1.2), 92.1 – 96.7	1.3 E-03
**Visual field mean deviation (dB)**	148	−2.9 (0.4), −3.8 – −2.0	126	−1.9 (0.4), −2.7 – −1.0	0.01
B. Excluding definite and probable glaucoma					
Trait	Highest PRS decile	Highest PRS decile	Lowest PRS decile	Lowest PRS decile	P-value
	N	(SE), 95% CI	N	(SE), 95% CI	
**Goldman IOP (mmHg)**^1^	200	15.2 (0.3), 14.5 – 15.8	225	13.6 (0.3), 13.1 – 14.2	3.0 E-06
**Corneal compensated IOP (mmHg)**^1^	201	16.6 (0.3), 16.0 – 17.3	227	14.8 (0.3), 14.3 – 15.4	1.7 E-07
**Global Circumpapillary RNFL thickness (μm)**	213	95.0 (1.1), 92.8 – 97.2	226	96.1 (1.0), 94.1 – 98.1	0.30
**Visual field mean deviation (dB)**	129	−1.8 (0.4), −2.6 – −1.0	121	−1.3 (0.3), −2.0 – −0.6	0.16

Adjusted means (± SE) with 95% confidence intervals for clinical traits by highest vs. lowest PRS deciles. Differences were assessed using linear regression adjusted for age, gender, and ancestry, with significance tested via pairwise comparisons of estimated marginal means. Possible glaucoma cases were included as controls.

1 Patients taking IOP-lowering medications are excluded.

2 Patients with low image quality are excluded.

3 Patients with unreliable visual fields are excluded. Abbreviations: PRS=polygenic risk score; 95% CI=95% confidence interval; IOP=intraocular pressure; RNFL=retinal nerve fiber layer.
